# Inhibition of pulmonary nuclear factor kappa-B decreases the severity of acute *Escherichia coli *pneumonia but worsens prolonged pneumonia

**DOI:** 10.1186/cc12696

**Published:** 2013-04-27

**Authors:** James Devaney, Gerard F Curley, Mairead Hayes, Claire Masterson, Bilal Ansari, Timothy O'Brien, Daniel O'Toole, John G Laffey

**Affiliations:** 1 Regenerative Medicine Institute, National University of Ireland Galway, University Road, Newcastle, Galway, Ireland; 2 Department of Anaesthesia and Critical Care, School of Medicine, Clinical Sciences Institute, National University of Ireland Galway, University Road, Newcastle, Galway, Ireland; 3 Department of Anesthesia, Keenan Research Centre in the Li Ka Shing Knowledge Institute, St. Michael's Hospital, University of Toronto, 30 Bond Street, Toronto, ONT, M5B 1W8, Canada

**Keywords:** Acute lung injury, inhibitory kappa-B alpha, rat, acute respiratory distress syndrome, bacteria, pneumonia, gene therapy

## Abstract

**Introduction:**

Nuclear factor (NF)-κB is central to the pathogenesis of inflammation in acute lung injury, but also to inflammation resolution and repair. We wished to determine whether overexpression of the NF-κB inhibitor IκBα could modulate the severity of acute and prolonged pneumonia-induced lung injury in a series of prospective randomized animal studies.

**Methods:**

Adult male Sprague-Dawley rats were randomized to undergo intratracheal instillation of (a) 5 × 10^9 ^adenoassociated virus (AAV) vectors encoding the IκBα transgene (5 × 10^9 ^AAV-IκBα); (b) 1 × 10^10 ^AAV-IκBα; (c) 5 × 10^10 ^AAV-IκBα; or (d) vehicle alone. After intratracheal inoculation with *Escherichia coli*, the severity of the lung injury was measured in one series over a 4-hour period (acute pneumonia), and in a second series after 72 hours (prolonged pneumonia). Additional experiments examined the effects of IκBα and null-gene overexpression on *E. coli*-induced and sham pneumonia.

**Results:**

In acute pneumonia, IκBα dose-dependently decreased lung injury, improving arterial oxygenation and lung static compliance, reducing alveolar protein leak and histologic injury, and decreasing alveolar IL-1β concentrations. Benefit was maximal at the intermediate (1 × 10^10^) IκBα vector dose; however, efficacy was diminished at the higher (5 × 10^10^) IκBα vector dose. In contrast, IκBα worsened prolonged pneumonia-induced lung injury, increased lung bacterial load, decreased lung compliance, and delayed resolution of the acute inflammatory response.

**Conclusions:**

Inhibition of pulmonary NF-κB activity reduces early pneumonia-induced injury, but worsens injury and bacterial load during prolonged pneumonia.

## Introduction

Acute lung injury (ALI) and acute respiratory distress syndrome (ARDS) are life-threatening disorders, for which no specific therapy is known. When ARDS occurs in the setting of multisystem organ failure, mortality rates more than 60% have been reported, with significant morbidity in 50% of survivors [1]. ALI and ARDS develop most commonly in the context of severe sepsis [2], particularly infection with gram-negative bacilli such as *Escherichia coli *(*E. coli*) [3], and sepsis-induced ARDS has the worst outcome [4].

Nuclear factor kappa B (NF-κB) is a key transcriptional regulator in the setting of inflammation and injury and plays a role in diverse inflammatory disorders, including acute lung injury [5]. Activation of NF-κB occurs in response to diverse stimuli, such as endotoxin, which bind to cell-surface receptors that in turn activate the canonic and/or noncanonic signaling pathway. This signaling cascade ultimately results in the phosphorylation and inactivation of the cytosolic inhibitor IκB complex, which then dissociates, allowing NF-κB to translocate to the nucleus to initiate gene transcription [5]. Inhibition of NF-κB reduces injury in preclinical models of ALI, including ischemia-reperfusion [6], endotoxemia [7], and cecal ligation and puncture-induced sepsis [8]. However, NF-κB also exerts important cytoprotective effects, promoting cell survival, resolution of inflammation, and wound repair [9]. Of interest, NF-κB signaling plays a central role in the host response to lung bacterial infection [10]. Consequently, inhibition of NF-κB may constitute a double-edged sword, particularly in pneumonia-induced ALI/ARDS, in which immune competence is essential to eradication of the infectious agent [11].

We wished to determine the potential for inhibition of pulmonary NF-κB activity to modulate the severity of pneumonia-induced lung injury. We used a gene-based therapy approach, via intrapulmonary delivery of three different doses of adenoassociated viral vector encoding the NF-κB inhibitor *IκBα *gene (AAV-IκBα), to modulate the NF-κB signaling pathway in the lung. We hypothesized that pulmonary overexpression of the NF-κB inhibitor IκBα would (a) attenuate the severity of the lung injury induced by acute *E. coli *pneumonia; but would (b) worsen the severity of prolonged *E. coli *pneumonia-induced lung injury; and (c) a dose-response relation would exist, with higher AAV-IκBα doses having the greatest effect.

## Materials and methods

Specific-pathogen-free adult male Sprague-Dawley rats (350 to 450 g) were used in all studies. The experimental model was based on those previously reported [12-14]. All work was approved by the National University of Ireland Galway Research Ethics Committee and conducted under license from the Department of Health, Ireland.

### Preparation of AAV vectors

AAV-vector production was carried out as previously described, with several modifications [15]. The *IκBα-SuperRepressor (IκBα-SR*) gene (1,566 bp) was ligated into the pAAV-MCS vector (Agilent Technologies Inc., Santa Clara, CA, USA), and plasmid size confirmed by gel electrophoresis and validated by sequencing (Eurofins MWG Operon, Ebersberg, Germany). The *IκBα-*FLAG plasmid DNA and AAV serotype 6 envelope were generated and sent to Virapur for AAV production (VIRAPUR, 6160 Lusk Blvd., Suite C-101, San Diego, CA, USA). AAV serotype 6 was chosen because AAV has reduced immunogenicity, the virus plasmid size is sufficient for the *IκBα-SuperRepressor (IκBα-SR) *gene, and serotype 6 has demonstrated tropism for lung epithelial cells.

Viral vector particle titers were determined with quantitative real-time polymerase chain reaction (qRT-PCR) and aliquoted and stored at -80^o^C. As required, an aliquot was thawed and added to 75 μl of the porcine surfactant Curosurf (120 mg/ml) (Trinity-Chiesi Pharmaceuticals Limited, Cheadle, UK), and a final instillate volume of 300 μl was made up with PBS. For those animals receiving vehicle only, the instillate was 75 μl of Curosurf mixed with 225 µl of phosphate-buffered saline (PBS). Curosurf surfactant was added to each instillate, as it was demonstrated in prior studies to enhance spread and improve transgene expression [16].

### Acute and prolonged *E. coli*-induced lung injury

#### Experimental design

Animals were randomized to intratracheal instillation of the following: (a) vehicle alone; (b) 5 × 10^9 ^drp AAV *IκBα-SR*; (c) 1 × 10^10 ^drp AAV *IκBα-SR*; or (d) 5 × 10^10 ^drp AAV *IκBα-SR*, and to undergo subsequent acute (Series 1) or prolonged (Series 2) pneumonia-induced ALI. Additional experimental series examined the effects of 1 × 10^10 ^AAV-*IκBα *versus 1 × 10^10 ^AAV-Null in both acute and prolonged *E. coli *and sham pneumonia.

#### Vector instillation

Animals were anesthetized by inhalational induction with isoflurane and an intraperitoneal injection of 40 mg/kg ketamine (Pfizer, Kent, UK). After confirmation of depth of anesthesia, laryngoscopy was performed (Welch Allyn Otoscope; Buckinghamshire, UK), and the trachea intubated with a size 16 intravenous catheter (BD Insyte; Becton Dickinson Ltd., Oxford, UK). After instillation of vector or vehicle, depending on the specific series, animals were extubated and allowed to recover from anesthesia.

#### *E. coli*/vehicle instillation

The *E. coli *used in these experiments is E5162 (serotype: O9 K30 H10) and was supplied by the National Collection of Type Cultures, Central Public Health Laboratory, London, England. The *E. coli *were stored on preservative beads (Protect, Lancashire, England) at -80°C. Beads were placed in 3-ml vials of peptone water (Cruinn Diagnostics, Dublin, Ireland) and incubated at 37°C for 18 to 24 hours to allow bacterial concentrations to reach a plateau. The bacterial suspension was then centrifuged and washed in phosphate-buffered saline to produce the inoculum. The bacterial load in each inoculum was determined by plating serial dilutions on agar plates. Preliminary experiments were performed to determine the bacterial load of intratracheal *E. coli *required to produce a lung injury over a 4-hour and over a 72-hour period.

#### Acute pneumonia protocol

Ninety-six hours after virus instillation, animals were anesthetized with intraperitoneal 80 mg/kg ketamine and 8 mg/kg xylazine, and anesthesia maintained with Alfaxalone (Alfaxadone 0.9% and alfadadolone acetate 0.3%). A tracheostomy tube was inserted, and intraarterial access was sited in the carotid artery. Muscle relaxation was induced with cisatracurium besylate, and the lungs were mechanically ventilated with 30% O_2 _in 70% N_2_. After 20 minutes, arterial blood gas measurement was performed and 1 × 10^11 ^*E. coli *in a 300-μl PBS suspension (or vehicle alone) instilled via the tracheostomy [12,13]. Animals were ventilated for 4 hours, with systemic arterial blood pressure, peak airway pressure, and body temperature continually measured. Lung compliance and arterial blood gas analysis was measured hourly, and body temperature was maintained at 36°C to 37.5°C.

#### Prolonged pneumonia protocol

Animals were anesthetized by inhalational induction with isoflurane and intraperitoneal 40-mg/kg ketamine (Pfizer, Kent, UK). After confirmation of anesthesia depth, 5 × 10^9 ^*E. coli *in a 300-μl PBS suspension was instilled into the trachea under direct vision, and the animals allowed to recover [17]. Animals were monitored closely for 72 hours after *E. coli *instillation, and then reanesthetized, tracheostomized, and mechanical ventilation was instituted, and injury severity assessed, as described [18].

### Postmortem analyses

At the end of the protocols, the animals were killed by exsanguination under anesthesia, and the plasma snap-frozen for later analysis. The heart-lung block was dissected from the thorax, bronchoalveolar lavage (BAL) was performed, and BAL fluid differential leukocyte counts and lung bacterial colony counts were completed. BAL fluid was centrifuged, and the supernatant was snap-frozen and stored at -80°C. BAL concentrations of IL-1β, TNF-a, IL-6, CINC-1, IL-10, and KGF were determined by using ELISA (R&D Systems, Abingdon, UK), and BAL protein concentrations were measured (Micro BCA Protein assay kit; Pierce, Rockford, IL, USA) [16]. The left lung was isolated and fixed, and the extent of histologic lung damage determined by using quantitative stereologic techniques [18].

### Assessment of transgene expression and efficacy

*IκBα-FLAG *transgene expression was determined in lung homogenates with real-time PCR and Western blotting, as previously described [16,19]. In brief, RNA was extracted from lung tissue, cDNA was synthesised, and quantitative PCR was performed for *IκBα-FLAG*, normalized against a *GAPDH *control product. For Western blot IκBα-FLAG analysis, total cell protein was extracted, protein concentration was determined, and samples were electrophoresed on an SDS-PAGE gel and transferred to nitrocellulose [20]. Primary anti-human IκBα-FLAG monoclonal antibody (Sigma-Aldrich, St. Louis, MO, USA) was used, with secondary antibody conjugated to horseradish peroxidase (Cell Signaling Technology, Danvers, MA, USA), and the membrane incubated with a chemiluminescent substrate (SuperSignal West Pico; Pierce).

The effect of *IκBα *overexpression on the activation of the NF-κB pathway was assessed by measurement of nuclear accumulation of the activated P65 subunit of NF-κB [19]. Nuclear extracts were performed on homogenized rat lung tissue by using a NE-PER Nuclear and Cytoplasmic Extraction Kit (Fisher Scientific Ireland, Dublin, Ireland), and NF-κB (p65) measured by using an NF-κB Transcription Factor Assay Kit (Cayman Chemical Company, Ann Arbor, MI, USA).

### Data presentation and analysis

Continuous responsive variables are summarized by using mean (SD) and median (interquartile range, IQR) as necessary. The proportion of animals surviving was analyzed by using the χ^2 ^test. All other data were analyzed with one-way ANOVA, followed by the Dunnett test or by Kruskal-Wallis, followed by the Dunn test, with the vehicle group used as the reference group for all comparisons. The assumptions underlying all models were checked by using suitable residual plots. A *P *value of <0.05 was considered statistically significant.

## Results

### Acute pneumonia-induced ALI

Sixty-eight animals were entered into these experimental series. In the first set of experiments, 48 animals were randomized to receive (a) vehicle alone (*n *= 12); (b) 5 × 10^9 ^AAV-IκBα *n *= 12); (c) 1 × 10^10 ^AAV-IκBα (*n *= 12); (d) 5 × 10^10 ^AAV-IκBα (*n *= 12), and all animals subsequently underwent intratracheal *E. coli *instillation. No differences were noted between the groups at baseline (Table 1).

**Table 1:** Data regarding animals subjected to acute pneumonia-induced ALI

Variable	Vehicle	5 × 10^9 ^IκBα	1 × 10^10 ^IκBα	5 × 10^10 ^IκBα
Number of animals	12	12	12	12

Animal weight (g)	456 ± 55	486 ± 54	469 ± 52	438 ± 86

Bacteria instilled (CFU × 10^10^)	1.1 ± 0.2	2.1 ± 0.2	2.6 ± 2.0	2.1 ± 2.2

Baseline arterial PO_2 _(kPa)	18.7 ± 0.5	18.7 ± 0.8	18.8 ± 0.8	18.7 ± 0.8

Baseline static compliance (ml/mm Hg)	0.93 ± 0.1	0.95 ± 0.1	0.99 ± 0.2	0.87 ± 0.2

Animal survival (number, %)	4 (33)	7 (58)	11 (92)*	5 (42)

Duration of animal survival (hours)	2.75(2, 4)	4 (1.5, 4)	4 (4, 4)*	3 (1.75, 4)

Lung homogenate bacterial counts (CFU × 10^9^)	0.8 (0.4, 1.7)	1.4 (0.2, 2.6)	0.6 (0.4, 0.9)	0.7 (0.4, 0.9)

BAL neutrophils (× 10^7^/ml)	5.9 (1.5, 8.4)	3.7 (1.5, 8.9)	6.2 (1.8, 6.7)	8.5 (1.1, 9.1)

BAL mononuclear cells (× 10^7^/ml)	2.2 (1.4, 9.1)	0.57 (2.7, 7.0)	1.5 (1.2, 9.3)	1.8 (1.1, 9.6)

Note: Data are expressed as mean ± SD. *Significantly different from Vehicle group.

**Figure 1:**
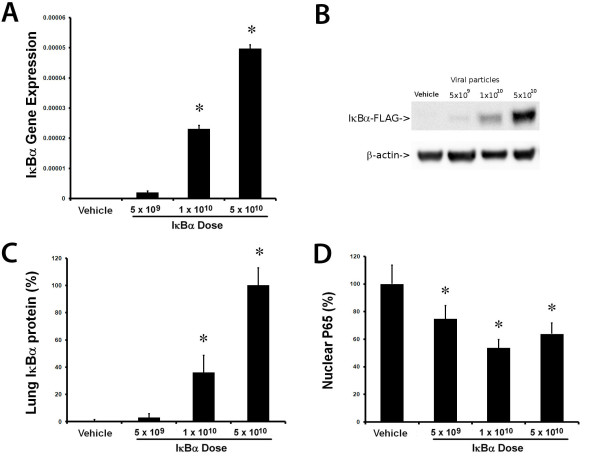
**Overexpression of IκBα-reduced NF-κB activity**. Delivery of the adeno-associated viral vector encoding IκBβ resulted in a dose-dependent increase in lung homogenate IκBβ FLAG mRNA expression **(A) **and IκBβ protein concentrations, as demonstrated by the representative Western blot **(B)**, and by densitometry of Western blots (*n *= 3 per group) **(C)**. IκBβ decreased *Escherichia coli*-induced nuclear accumulation of the activated P65 subunit of NF-κB **(D)**. Vehicle, animals that received intratracheal vehicle alone; IκBβ 5 × 10^9^, animals that received 5 × 10^9 ^AAV6 particles encoding IκBβ; IκBβ 1 × 10^10^, animals that received 1 × 10^10 ^AAV6 particles encoding IκBβ; IκBβ 5 × 10^10^, animals that received 5 × 10^10 ^AAV6 particles encoding IκBβ. *Significantly different from vehicle group (*P *< 0.05, ANOVA).

IκBα expression and function Pulmonary instillation of AAV-*IκBα *produced a dose-dependent increase in lung *IκBα *gene transcription (Figure 1A) and protein production (Figure 1B, C). Densitometry of IκBα-FLAG Western blot (*n *= 3 per group) confirmed this dose-dependent increase in IκBα protein (Figure 1C). *IκBα *overexpression decreased *E. coli*-induced NF-κB activation, as measured by nuclear accumulation of the activated P65 subunit of NF-κB (Figure 1D).

Survival and bacterial load IκBα overexpression enhanced animal survival, with survival rates of 33% in the Vehicle group, 58% with 5 × 10^9 ^*IκBα*, and 92% with 1 × 10^10 ^*IκBα *Of concern, the highest *IκBα *(5 × 10^10^) dose abolished the survival benefit (Table 1). *IκBα *overexpression also enhanced the duration of animal survival, which followed this same pattern, with the effect greatest with the1 × 10^10 ^*IκBα *dose (Table 1). Importantly, IκBα did not alter lung bacterial loads (Table 1).

#### IκBα modulated lung-injury severity

**Figure 2:**
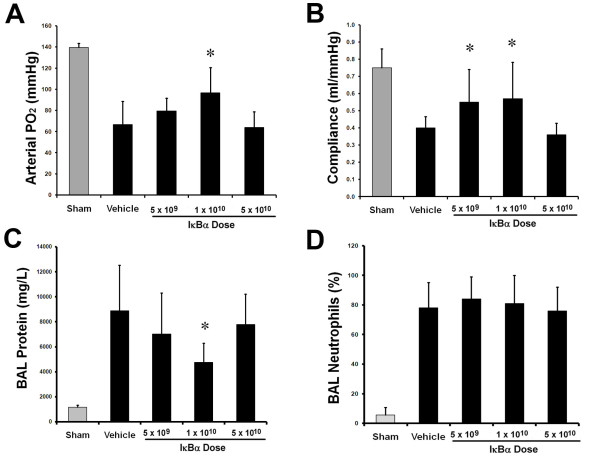
**IκBα modulated severity of acute pneumonia-induced ALI**. Overexpression of IκBα attenuated the decrement in arterial oxygenation **(A)**, and in static lung compliance **(B)**, and decreased BAL protein concentrations **(C)**, after *Escherichia coli*-induced ALI. Beneficial effects were greatest with the intermediate IκBα (1 × 10^10 ^particles) dose but were abolished by the higher IκBα (5 × 10^10 ^particles) dose. No effect of IκBα **was noted **on BAL neutrophil infiltration **(D)**. The gray bars represent sham (that is, uninfected) animals. Vehicle, animals that received intratracheal vehicle alone; IκBα 5 × 10^9^, animals that received 5 × 10^9 ^AAV6 particles encoding IκBα; IκBα 1 × 10^10^, animals that received 1 × 10^10 ^AAV6 particles encoding IκBα; IκBα 5 × 10^10^, animals that received 5 × 10^10 ^AAV6 particles encoding IκBα. Significantly different from vehicle group (*P *< 0.05, ANOVA).

**Figure 3:**
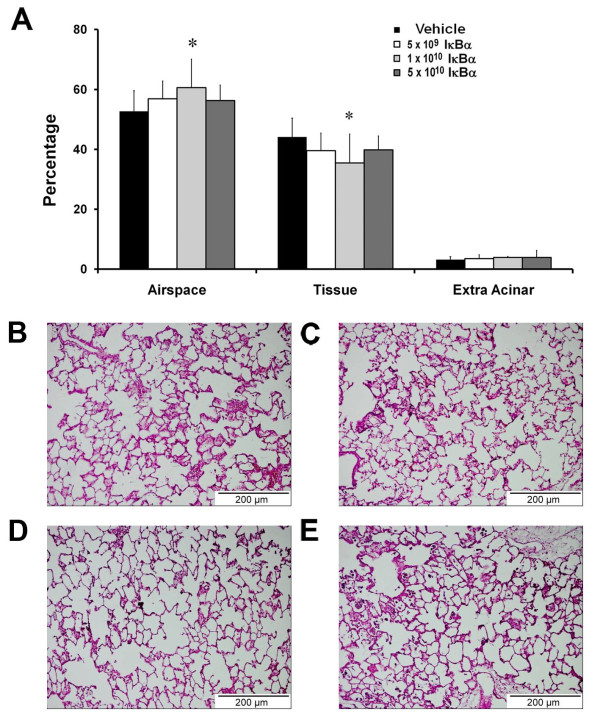
**IκBα modulated structural lung injury induced by acute pneumonia**. Overexpression of IκBα attenuated the decrement in alveolar airspace and the increase in alveolar tissue **(A) **after pneumonia-induced lung injury. **(B) **Image from a pneumonia-injured lung that received intratracheal vehicle, demonstrating increased wall thickness and inflammatory cell infiltrate. (**C **through **E) **Images from pneumonia-injured lungs that received increasing doses of intratracheal vector encoding the IκBα transgene. The animals that received the intermediate (5 × 10^10^) dose demonstrated reduced injury and inflammatory cell infiltration. Vehicle, animals that received intratracheal vehicle alone; IκBα 5 × 10^9^, animals that received 5 × 10^9 ^AAV6 particles encoding IκBα; IκBα 1 × 10^10^, animals that received 1 × 10^10 ^AAV6 particles encoding IκBα; IκBα 5 × 10^10^, animals that received 5 × 10^10 ^AAV6 particles encoding IκBα. Significantly different from vehicle group (*P *< 0.05, ANOVA). **S**cale bar, 200 μm.

The *E. coli *instillation induced a severe acute lung injury (Figure 2). *IκBα *overexpression reduced the *E. coli*-induced decrement in arterial oxygenation, with benefit greatest at intermediate (1 × 10^10^) *IκBα *vector dose, but abolished at the higher (5 × 10^10^) *IκBα *dose (Figure 2A and Table 1). A similar pattern was seen with regard to the decrement in static compliance (Figure 2B), and pulmonary permeability, as assessed by protein leak into the BAL fluid (Figure 2C), but no effect was seen in regard to BAL neutrophil or mononuclear cell counts (Figure 2D and Table 1). *IκBα *reduced *E. coli*-induced histologic evidence of lung injury, with the intermediate (1 × 10^10^) vector dose of *IκBα *again most effective (Figure 3A through E).

#### IκBα modulates pulmonary inflammation

**Figure 4:**
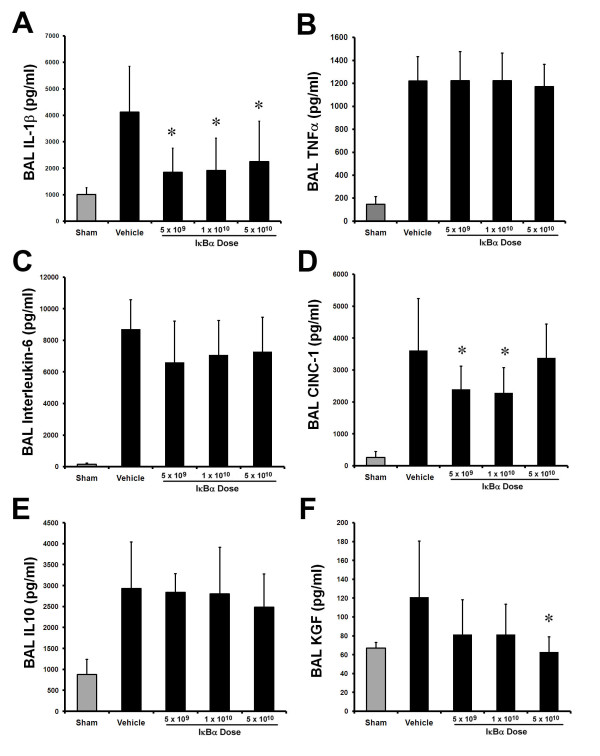
**IκBα dose-dependently modulated the inflammatory response to acute pneumonia-induced ALI**. Overexpression of IκBα attenuated the increase in BAL interleukin-1β **(A)**, but did not alter BAL TNF-α **(B)**, or interleukin-6 concentrations **(C)**, after *Escherichia coli*-induced ALI. IκBα dose dependently decreased BAL CINC-1 **(D)**, did not alter BAL IL-10 concentrations **(E)**, and dose dependently decreased BAL KGF concentrations **(F)**. These effects were greatest with the highest IκBα (5 × 10^10 ^particles) dose. The gray bars represent sham (uninfected) animals. Vehicle, animals that received intratracheal vehicle alone; IκBα 5 × 10^9^, animals that received 5 × 10^9 ^AAV6 particles encoding IκBα; IκBα 1 × 10^10^, animals that received 1 × 10^10 ^AAV6 particles encoding IκBα; IκBα 5 × 10^10^, animals that received 5 × 1010^9 ^AAV6 particles encoding IκBα, BAL, bronchoalveolar lavage; IL-1β, interleukin-1β; TNF-α, tumor necrosis factor-α; CINC-1, cytokine-induced neutrophil chemoattractant-1; KGF, keratinocyte growth factor. Significantly different from vehicle group (*P *< 0.05, ANOVA).

The *E. coli *instillation activated the inflammatory response. *IκBα *significantly decreased alveolar IL-1β, but did not alter BAL TNF-α or interleukin-6 concentrations (Figure 4A through C). *IκBα *dose-dependently modulated BAL CINC-1 and KGF, but did not alter BAL IL-10 concentrations (Figure 4D, F).

### Sham acute pneumonia and null-transgene series

Additional experiments were performed to determine the effect of overexpression of a null transgene (versus vehicle; *n *= 4 per group) on the severity of *E. coli *pneumonia, and to determine the effect of *IκBα *and null-gene overexpression in sham pneumonia (versus vehicle; *n *= 4 per group). In these studies, the null transgene did not alter the severity of *E. coli*-induced lung injury (Table 2). In addition, overexpression the null or *IκBα *transgenes in sham-injured animals did not alter lung function (Table 3).

**Table 2:** Overexpression of Null transgene does not modulate the severity of acute pneumonia-induced ALI

Variable	*E. coli *+ vehicle	*E. coli *+ null transgene
Number of animals	4	4

Animal weight (g)	443 ± 25	462 ± 33

Bacteria instilled (CFU × 10^10^)	1.1(0.9, 1.2)	1.2(0.5, 3.6)

Animal survival (*n*, %)	2 (50%)	2 (50%)

Duration of animal survival (hours)	3.5(2, 4)	3.75 (2.5, 4)

Lung homogenate bacterial counts (CFU × 10^9^)	1.7 (1.4, 3.8)	2.2 (1.2, 3.7)

Final arterial PO_2 _(kPa)	10.8 ± 0.8	9.3 ± 1.2

Final static compliance (ml/mm Hg)	0.39 ± 0.1	0.39 ± 0.1

BAL protein (mg/L)	7,203 (5,319, 7,646)	5,542 (2,994, 8,554)

BAL neutrophils (× 10^7^/ml)	3.8 (2.1, 5.2)	8.1 (4.6, 11.2)

BAL mononuclear cells (× 10^7^/ml)	0.88 (0.13, 0.72)	1.2 (0.18, 2.0)

Data are expressed as mean ± SD or median (interquartile range). Final data are data collected on completion of the experimental protocol. *Significantly different from Vehicle group. BAL, bronchoalveolar lavage; CFU, colony-forming unit.

**Table 3:** Overexpression of Null or IκBα transgenes does not alter lung function in animals subjected to sham pneumonia

Variable	Vehicle	IκBα transgene	Null transgene
Number of animals	4	4	4

Animal weight (g)	413 ± 30	426 ± 17	427 ± 19

Animal survival (n, %)	4 (100%)	4 (100%)	4 (100%)

Duration of animal survival (hours)	4 [4,4]	4 [4,4]	4 [4,4]

Final arterial PO_2 _(kPa)	18.6 ± 0.5	18.4 ± 1.1	19.9 ± 0.7

Final static compliance (ml/mm Hg)	0.65 ± 0.11	0.71 ± 0.17	0.58 ± 0.16

BAL protein (mg/L)	1197 [1061, 1296]	1353 [991, 2167]	1337 [950, 1869]

BAL neutrophils (× 10^4^/ml)	0.2 [0.0, 1.4]	1.3 [0.5, 1.8]	1.1 [0.2, 2.0]

BAL mononuclear cells (× 10^4^/ml)	21.0 [11, 22]	23 [10, 29]	14 [12, 18]

Data are expressed as mean ± SD or median (interquartile range). Final data are data collected on completion of the experimental protocol. *Significantly different from Vehicle group. BAL, bronchoalveolar lavage; CFU, colony-forming units.

### Prolonged pneumonia-induced ALI

Fifty-six animals were entered into these experimental series. In the first set of experiments, 44 animals were randomized to receive (a) vehicle (*n *= 11); (b) 5 × 10^9 ^AAV-IκBα (*n *= 11); (c) 1 × 10^10 ^AAV-IκBα (*n *= 11); (d) 5 × 10^10 ^AAV-IκBα (*n *= 11), and underwent prolonged *E. coli*-induced lung injury. No differences were found between the groups at baseline (Table 4).

**Table 4:** Data regarding animals subjected to prolonged pneumonia-induced ALI

Variable	Vehicle	5 × 10^9 ^IκBα	1 × 10^10 ^IκBα	5 × 10^10 ^IκBα
Number of animals	11	11	11	11

Animal weight (g)	383 ± 13	378 ± 19	413 ± 35	401 ± 47

Animal survival (number, %)	8 (73)	9 (82)	9 (82)	8 (73)

Duration of animal survival (hours)	65.5 ± 11.2	64.8 ± 16.2	63.3 ± 19.4	63.3 ± 16.1

Instilled *E. coli *bacterial load (CFU × 10^4^)	5.0 ± 1.0	5.0 ± 1.0	5.0 ± 1.0	5.0 ± 1.0

Peak airway pressure (mm Hg)	8.1 ± 1.4	6.5 ± 1.0	7.4 ± 1.0	8.0 ± 1.3

Lung homogenate bacterial counts (CFU × 10^4^)	1.1 (0.7, 3.1)	51.3 (3.1, 96)*	20.7 (15.9, 36.2)*	19.3 (16.5, 34.6)*

BAL neutrophils (× 10^9^/ml)	3.7 ± 3.3	2.3 ± 0.8	3.8 ± 2.2	1.7 ± 0.9

BAL mononuclear cells (× 10^9^/ml)	2.3 ± 1.8	0.4 ± 0.2*	0.9 ± 0.6*	0.4 ± 0.2*

Data are expressed as mean ± SD or median (interquartile range). Final data are data collected on completion of the experimental protocol. *Significantly different from Vehicle group. BAL, bronchoalveolar lavage; CFU, colony-forming units.

#### Animal Survival and Bacterial Load

IκBα did not alter the number or duration of animal survival (Table 4). However, IκBα substantially increased lung *E. coli *bacterial loads at all three IκBα doses (Table 4).

#### IκBα worsens lung injury

**Figure 5:**
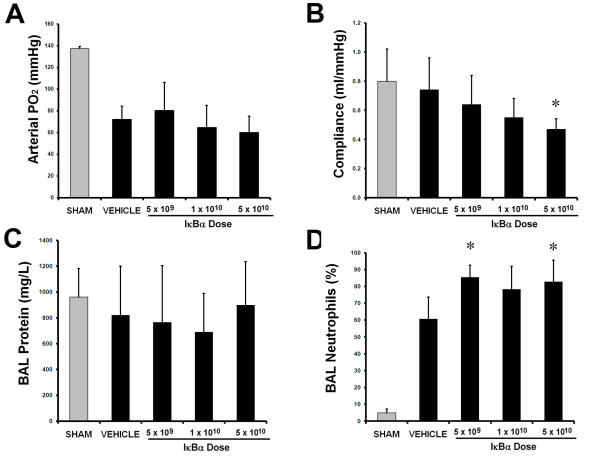
**IκBα worsened ALI induced by prolonged pneumonia**. Overexpression of IκBα did not alter the decrement in arterial oxygenation **(A)**. IκBα worsened the *Escherichia coli*-induced decrement in static lung compliance, with effect greatest with 5 × 10^10 ^IκBα particles **(B)**. IκBα did not alter BAL protein concentrations **(C)**, but did significantly increase the percentage of neutrophils in the alveolar infiltrate **(D)**. The gray bars represent sham (uninfected) animals. Vehicle, animals that received intratracheal vehicle alone; IκBα 5 × 10^9^, animals that received 5 × 10^9 ^AAV6 particles encoding IκBα; IκBα 1 × 10^10^, animals that received 1 × 10^10 ^AAV6 particles encoding IκBα; IκBα 5 × 10^10^, animals that received 5 × 10^10 ^AAV6 particles encoding IκBα. Significantly different from vehicle group (*P *< 0.05, ANOVA).

**Figure 6:**
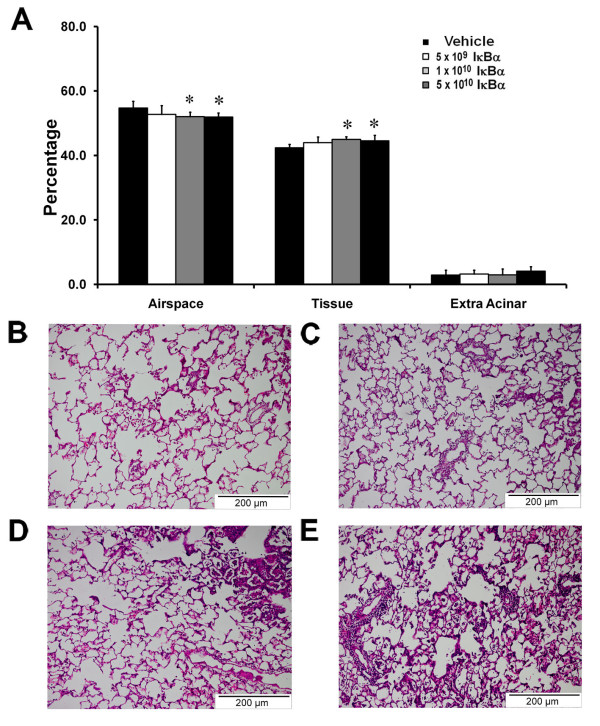
**IκBα worsened structural lung injury induced by prolonged pneumonia**. Overexpression of IκBα worsened the decrement in alveolar airspace and the increase in alveolar tissue **(A) **after pneumonia-induced lung injury. **(B) **Image from a prolonged pneumonia-injured lung that received intratracheal vehicle, demonstrating increased wall thickness and inflammatory cell infiltrate. **(C **through **E) **Images from a pneumonia-injured lung that received increasing doses of intratracheal vector encoding the IκBα transgene. The animals that received 1 to 5 × 10^10 ^doses demonstrated greater injury. Vehicle, animals that received intratracheal vehicle alone; IκBα 5 × 10^9^, animals that received 5 × 10^9 ^AAV6 particles encoding IκBα; IκBα 1 × 10^10^, animals that received 1 × 10^10 ^AAV6 particles encoding IκBα; IκBα 5 × 10^10^, animals that received 5 × 10^10 ^AAV6 particles encoding IκBα. *Significantly different from vehicle group (*P *< 0.05, ANOVA). Scale bar, 200 μm.

*E. coli *instillation induced a severe lung injury compared with sham pneumonia (Figure 5). IκBα overexpression did not alter the decrement in arterial oxygenation (Figure 5A). IκBα overexpression dose dependently worsened static lung compliance (Figure 5B). IκBα did not alter alveolar protein leak (Figure 5C), but did increase the proportion of neutrophils in the alveolar infiltrate (Figure 5D), while decreasing alveolar mononuclear cells (Table 4). IκBα worsened *E. coli-*induced histologic injury, with the intermediate (1 × 10^10^) and higher (5 × 10^10^) dose of IκBα, resulting in significantly greater injury (Figure 6A through E).

#### IκBα modulates pulmonary inflammation

**Figure 7:**
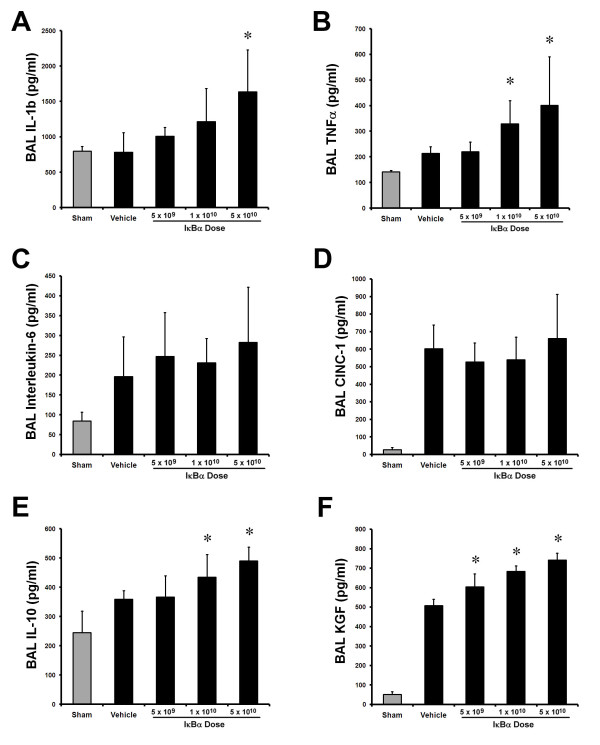
**IκBα dose-dependently modulated the inflammatory response to prolonged pneumonia**. Overexpression of IκBα dose-dependently increased BAL interleukin-1β **(A) **and TNF-α **(B) **concentrations compared with vehicle. No effect of IκBα was seen on interleukin-6 **(C) **or CINC-1 **(D) **concentrations. IκBα dose-dependently increased BAL IL-10 **(E)**, and KGF concentrations **(F)**. These effects were greatest with the highest IκBα (5 × 10^10 ^particles) dose. The gray bars represent sham (uninfected) animals. Vehicle, animals that received intratracheal vehicle alone; IκBα 5 × 10^9^, animals that received 5 × 10^9 ^AAV6 particles encoding IκBα; IκBα 1 × 10^10^, animals that received 1 × 10^10 ^AAV6 particles encoding IκBα; IκBα 5 × 10^10^, animals that received 5 × 10^10 ^AAV6 particles encoding IκBα. *Significantly different from vehicle group (*P *< 0.05, ANOVA).

Prolonged *E. coli *pneumonia activated the inflammatory response compared with sham pneumonia (Figure 7). IκBα overexpression dose-dependently increased alveolar IL-1β (Figure 7A) and TNF-α (Figure 7B), with concentrations highest after 5 × 10^10 ^IκBα. In contrast, no effect was seen on alveolar IL-6 (Figure 7C) or CINC-1 (Figure 7D) concentrations. IκBα increased alveolar IL-10 (Figure 7E) and KGF concentrations, with a step-wise dose effect seen (Figure 7F).

### Sham Prolonged Pneumonia

In a subsequent experiment, 12 animals were randomized to receive: (a) Vehicle (*n *= 4); (b) 1 × 10^10 ^AAV-Null (*n *= 4); and (3) 1 × 10^10 ^AAV-IκBα and to undergo subsequent sham (vehicle) instillation. The null or IκBα transgenes did not alter lung function in sham infected animals (Table 5).

**Table 5:** Overexpression of Null or IκBα transgenes does not alter lung function or activate the immune response in animals subjected to sham prolonged pneumonia-induced ALI

Variable	Vehicle	IκBα transgene	Null transgene
Number of animals	4	4	4

Animal weight (g)	411 ± 24	409 ± 14	395 ± 11

Animal survival (n, %)	4 (100%)	4 (100%)	4 (100%)

Duration of animal survival (hours)	72 (72, 72)	72 (72, 72)	72 (72, 72)

Final arterial PO_2 _(kPa)	18.3 ± 0.3	17.6 ± 2.5	18.3 ± 0.2

Final static compliance (ml/mm Hg)	0.71 ± 0.07	0.78 ± 0.09	0.75 ± 0.12

BAL protein (mg/L)	961 ± 221	829 ± 226	826 ± 180

BAL neutrophils (× 10^3^/ml)	1.1 ± 0.7	2.2 ± 1.7	1.4 ± 1.2

BAL mononuclear cells (× 10^4^/ml)	5.5 ± 3.6	5.2 ± 3.9	3.8 ± 2.4

Data are expressed as mean ± SD or median [interquartile range]. Final data are data collected on completion of the experimental protocol. *Significantly different from Vehicle group. BAL, bronchoalveolar lavage; CFU, colony-forming units

## Discussion

Pulmonary overexpression of IκBα attenuated acute pneumonia-induced lung injury, decreasing the severity of the decrement in lung function, whereas also decreasing the inflammatory response. In contrast, IκBα worsened lung injury and inflammation induced by prolonged pneumonia, increasing lung bacterial load and delaying resolution of the inflammatory response. These findings provide novel insights into the effects of NF-κB in pneumonia-induced lung injury, and raise concerns regarding therapeutic potential of inhibiting NF-κB, particularly in prolonged untreated pneumonia.

### E. coli acute and prolonged pneumonia

Infection with gram-negative bacilli such as *E. coli *is the commonest cause of ARDS [21,22] and is also a very common complication of ARDS due to other causes [23]. We studied *E. coli*-induced pneumonia, a well-characterized animal model that mimics the clinical development of ARDS very closely [23-28]. Intratracheal instillation of 1 × 10^11 ^*E. coli *resulted in physiological and pathologic changes consistent with a severe lung injury over a 6-hour period, similar to that previously reported [12,13]. Instillation of a lower dose of 5 × 10^9 ^*E. coli *produced a more gradually evolving injury over a 72-hour period, as previously described [14,17].

### NF-κB: role in lung inflammation

The role of the NF-κB signaling in the host immune response to lung injury is increasingly well understood [19]. NF-κB activation pathway gene polymorphisms alter the susceptibility to [29] and severity [30] of clinical ARDS. NF-κB is a dimer of a number of related proteins, including RelA (also known as P65), p50, p52, RelB, and cRel, with the RelA and p50 heterodimer, the most common form. On cell activation, by stimuli such as gram-negative bacterial endotoxin, diverse signaling pathways are activated, which converge to phosphorylate and activate the IκB kinase complex proteins (IKK), which then phosphorylate and inactivate the IκB proteins, which dissociates from NF-κB. The active NF-κB translocates to the nucleus and binds to specific cognate-binding sequences in the promoter or enhancer regions of different target genes to initiate transcription [5].

The therapeutic potential of strategies to inhibit NF-κB is evident from the demonstration that NF-κB inhibition decreases injury in nonseptic ALI models, including pulmonary [6] and systemic reperfusion, and endotoxemia [7]. Pulmonary overexpression of the RelB member of the NF-κB family decreases cigarette smoke-induced lung injury [31].

### ARDS, pneumonia, and NF-κB

The effects of modulation of the NF-κB in the setting of lung bacterial infection are less well understood [19]. NF-κB decoy oligodeoxynucleotides reduce ALI in mice in the early phases of cecal ligation and puncture-induced sepsis [8]. Selective inhibition of vascular endothelial NF-κB activity in endotoxemic transgenic mice reduced lung inflammation and increased survival [32]. Of interest, this approach improved survival and systemic organ function, but did not alter bacterial clearance, in septic mice [32]. However, others have found inhibition of NF-κB signaling to exert detrimental effects in the setting of infection. Inhibition of hepatocyte NF-κB activity reduced *Listeria monocytogenes *clearance, decreasing murine survival [11]. In contrast, clearance of pseudomonas bacteria from the mouse lung was enhanced by pulmonary overexpression of the RelA subunit of NF-κB [33], suggesting that the NF-κB pathway plays a pivotal role in maintaining immune competence and is essential to eradication of the infectious agent [11].

### NF-κB: role in lung inflammation, injury, and repair

NF-κB also promotes cell survival, resolution of inflammation, and repair after injury. Inhibition of NF-κB signaling retards pulmonary [20] and intestinal [34] epithelial wound healing. Maturational differences in lung NF-κB activation profiles exist, with NF-κB activation protecting the lung against hyperoxia [35] and endotoxemia [36] in neonatal rats. Consequently strategies to inhibit NF-κB, particularly if not spatially or temporally targeted, may have detrimental effects.

### Targeting pulmonary NF-κB

We wished to determine whether inhibition of pulmonary NF-κB activity could modulate the severity of pneumonia-induced lung injury. We found that pulmonary overexpression of the *IκBα *gene did reduce acute pneumonia-induced injury. IκBα decreased the decrement in arterial oxygenation and lung static compliance, decreased alveolar protein leak, and decreased histologic injury, compared with vehicle. IκBα modulated the cytokine response to *E. coli *instillation, decreasing alveolar IL-1β but increasing CINC-1 concentrations. Of importance, these effects were dose dependent, with benefit maximal at the intermediate (1 × 10^10^) *IκBα *dose, and a loss of efficacy at the higher (1 × 10^10^) *IκBα *concentration.

In contrast, in prolonged untreated *E. coli *pneumonia, pulmonary *IκBα *gene overexpression worsened the lung injury, and increased lung bacterial load. IκBα delayed the resolution of the acute inflammatory response, increasing the proportion of alveolar neutrophils while decreasing alveolar mononuclear cells, which comprise lymphocytes and macrophages and are considered important to repair. IκBα increased alveolar concentrations of TNF-α and IL-1β, cytokines implicated in the early phase of the response to septic insult. IκBα increased alveolar IL-10 concentrations, which may partly explain the ineffective response to the bacterial insult.

### NF-κB and sepsis: an integrated paradigm

The contrasting effects of NF-κB inhibition in acute versus prolonged pneumonia generate a number of important insights. First, the finding that NF-κB inhibition reduced the severity of acute pneumonia and decreased the host response induced ALI suggests that any benefit in this setting is mediated via a decreased host response to the instillation of *E. coli *[37]. This is consistent with the concept that in the early phases of pneumonia, secreted toxins, and the host immune response may be the predominant source of injury [37,38]. This is supported by the fact that NF-κB inhibition is protective in nonseptic inflammatory ALI models. Second, this effect was dose dependent, with lower and intermediate IκBα doses protective and the beneficial effects ablated at higher doses. This suggests a U-shaped dose-effect curve, reducing the therapeutic utility of approaches to inhibit NF-κB in the setting of acute pneumonia. Third, IκBα worsened the severity of prolonged pneumonia, substantially increasing lung bacterial loads, and resulting in a persisting pulmonary inflammatory response. IκBα, by inhibiting the early host response to *E. coli *instillation, may have led to a persistence of infection, ultimately worsening lung injury. Finally, the beneficial effects of NF-κB inhibition in acute pneumonia were lost at the highest dose. The reasons for this is unclear, but may include NF-κB inhibition mediated reduction in lung epithelial repair [20], apoptosis of airway epithelial cells [16] and effects on lung macrophage function [39].

## Conclusions

IκBα overexpression reduces injury severity and inflammation in early pneumonia, but slows resolution of inflammation and bacterial clearance and worsens injury during prolonged pneumonia. These findings raise serious questions regarding the utility of strategies that inhibit the innate immune response, such as NF-κB, in live bacterial pneumonia.

**Key Message: **Inhibition of pulmonary NF-κB activity decreases the severity of early pneumonia-induced lung injury, but worsens injury severity and bacterial load during prolonged pneumonia.

## Abbreviations

AAV: adenoassociated virus; ALI: acute lung injury; ANOVA: analysis of variance; ARDS: acute respiratory distress syndrome; BAL: bronchoalveolar lavage; CINC-1: cytokine-induced neutrophil chemoattractant-1; DNA: deoxyribonucleic acid; *E. coli: Escherichia coli; *IκBα: inhibitory factor kappa B alpha; IL: interleukin; IQR: interquartile range; KGF: keratinocyte growth factor; qRT-PCR: quantitative real-time polymerase chain reaction; NF-κB: nuclear factor kappa B; PBS: phosphate-buffered saline; RNA: ribonucleic acid; TNF-α: tumor necrosis factor-α

## Competing interests

The authors declare that they have no competing interests.

## Authors' contributions

JD performed the animal experiments, assays, and histologic analyses, analyzed the data, drafted the manuscript, and agreed to the final submitted version. GFC and MH assisted with animal experiments, contributed to drafting the manuscript, and agreed to the final submitted version. CM, BA, and DOT performed assays and histologic analyses, contributed to drafting the manuscript, and agreed to the final submitted version. TOB conceived and designed the experiments, provided viral vector expertise, drafted the manuscript, and agreed to the final submitted version. JL conceived and designed the experiments, analyzed the data, drafted the manuscript, and agreed to the final submitted version.
